# Supplementary figures and images for: Robotic selection for the rapid development of stable CHO cell lines for HIV vaccine production
Source: PLoS One. 2018 Aug 2;13(8):e0197656. doi: 10.1371/journal.pone.0197656 (PMC6071959; doi:10.1371/journal.pone.0197656)

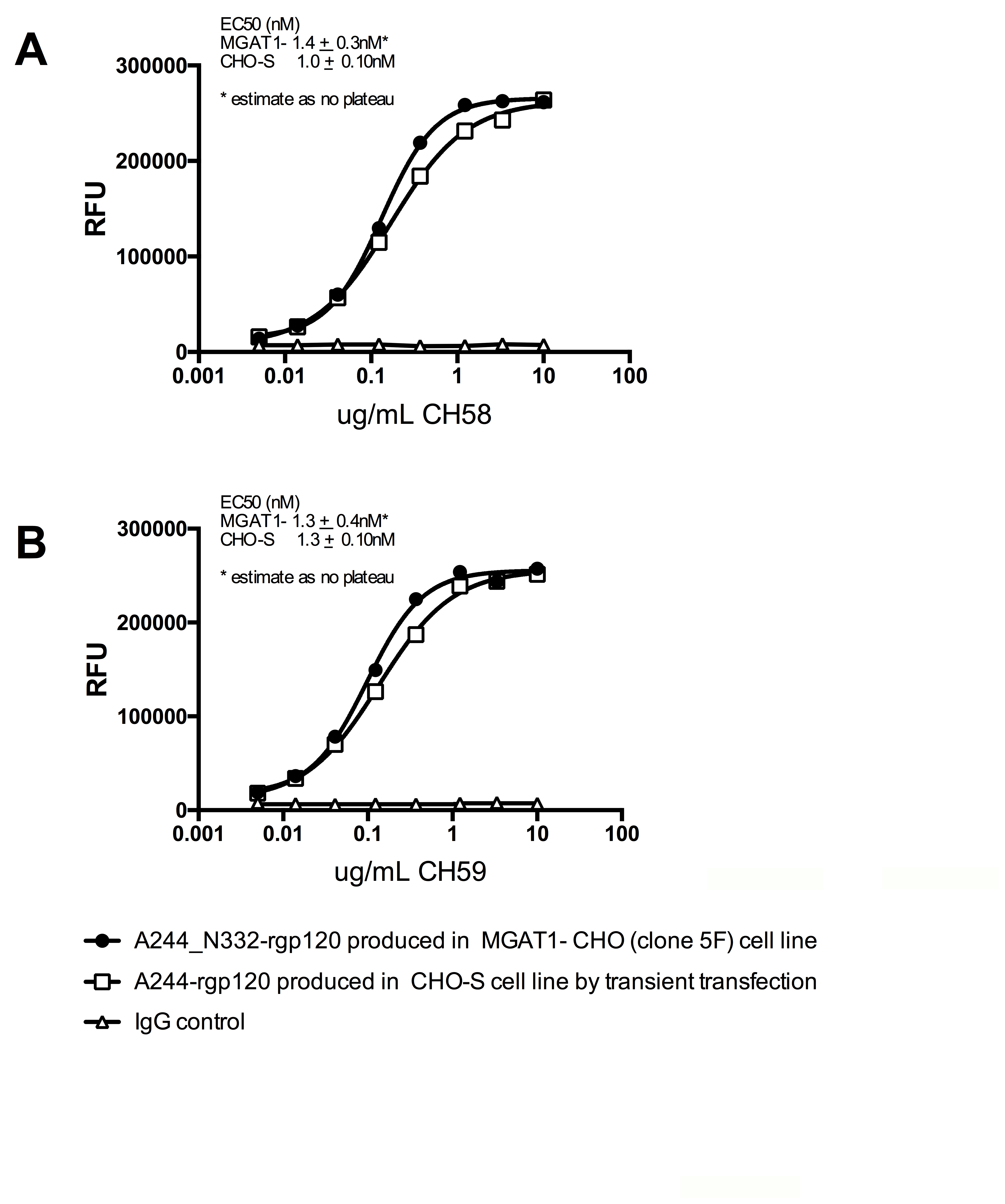

Supplement: S1 Fig — A244_N332-rgp120 was purified from the stable clone 5F MGAT1- CHO cell line (closed circles) and A244-rgp120 expressed and purified from transiently transfected CHO-S cells (open squares). Antibody binding of CH58 (A) or CH59 (B) was measured by a fluorescent immunoassay (FIA) and plotting using GraphPad prism 6 for Mac. (TIFF) [file pone.0197656.s001.tiff]
