# Supplementary material for: Robotic selection for the rapid development of stable CHO cell lines for HIV vaccine production
Source: PLoS One. 2018 Aug 2;13(8):e0197656. doi: 10.1371/journal.pone.0197656 (PMC6071959; doi:10.1371/journal.pone.0197656)
Supplement: S1 Table — The MGAT1- A244 N332 cell line was confirmed to be of Chinese hamster origin and no mammalian interspecies contamination was detected by Real-time PCR analysis. IDEXX BioResearch uses strict quality control and assurance measures in accordance with good laboratory practice. Microbiologic evaluation confirmed no fungal or bacterial growth. “+” indicates genetic confirmation “-”indicates absence of genetic sequence. (DOCX) [file pone.0197656.s002.docx]

| **Species** | **Result** |
| --- | --- |
| Mouse | - |
| Rat | - |
| Human | - |
| Chinese hamster | + |
| African green monkey | - |

**S1 Table. Interspecies and Sterility Testing** **by IDEXX laboratories Columbia Missouri.** The MGAT1- A244 N332 cell line was confirmed to be of Chinese hamster origin and no mammalian interspecies contamination was detected by Real-time PCR analysis. IDEXX BioResearch uses strict quality control and assurance measures in accordance with good laboratory practice. Microbiologic evaluation confirmed no fungal or bacterial growth. “+” indicates genetic confirmation “-“ indicates absence of genetic sequence.
