# Supplementary material for: Robotic selection for the rapid development of stable CHO cell lines for HIV vaccine production
Source: PLoS One. 2018 Aug 2;13(8):e0197656. doi: 10.1371/journal.pone.0197656 (PMC6071959; doi:10.1371/journal.pone.0197656)
Supplement: S2 Table — The MGAT1- A244 N332 cell line was assayed against the IMPACT2F and h-IMPACT Profile 1 by RT-PCR, “+” indicates a positive result in PCR assay. “-”a negative result. (DOCX) [file pone.0197656.s003.docx]

| **Pathogen** | **Result** |
| --- | --- |
| EBV | - |
| HAdV | - |
| HCMV | - |
| Hepatitis A | - |
| Hepatitis B | - |
| Hepatitis C | - |
| HHV 6 | - |
| HHV 8 | - |
| HIV1 | - |
| HIV2 | - |
| HPV16 | - |
| HPV18 | - |
| HSV 1 | - |
| HSV 2 | - |
| HTLV 1 | - |
| HTLV 2 | - |
| VZV | - |
| Ectromelia | - |
| EDIM | - |
| Hantaan | - |
| K virus | - |
| LCMV | - |
| LDEV | - |
| **Pathogen** | **Result** |
| MAV1 | - |
| MAV2 | - |
| mCMV | - |
| MHV | - |
| MNV | - |
| MTV | - |
| *Mycoplasma pulmonis* | - |
| *Mycoplasma sp.* | - |
| MVM | - |
| MPV | - |
| KRV | - |
| RMV | - |
| RPV | - |
| H1 | - |
| Polyoma | - |
| PVM | - |
| rCMV | - |
| RCV/SDAV | - |
| REO3 | - |
| Sendai | - |
| Seoul | - |
| Sin Nombre | - |
| Treponema pallidum | - |
| RTV | - |

**S2 Table. Pathogen Testing by IDEXX laboratories Columbia Missouri.** The MGAT1- A244 N332 cell line was assayed against the IMPACT2F and h-IMPACT Profile 1 by RT-PCR, “+” indicates a positive result in PCR assay. “-“ a negative result.

Real-Time PCR Assay Validation: All Real-Time PCR assays are put through an extensive validation process. Assays are designed utilizing all genome sequences deposited in GenBank or from sequence information generated in-house for organisms with limited or no genome sequences available from public databases. Assays are designed to be specific for the intended target by choosing genes and genome regions unique to the intended target. In addition, to ensure robust assay sensitivity, the most conserved genome regions of the intended target are chosen such that the primer and probe binding regions are areas less likely to be altered by potential genetic variation among field strains of the intended organism. Assays must pass analytical validation when tested in triplicate against dilutions of 1) a known positive control and 2) a known positive clinical case sample with the following criteria being met and reproduced on different run days: amplification efficiency of 95-105%, linearity over 5 points, calculated coefficient of variation (CV) of crossing points (Cp) equal to or smaller than 3%, CV calculated with absolute values equal to or smaller than 20%, r2 value equal to or larger than 0.993, signal to noise ratio of fluorescent signal: ≥10 and analytical sensitivity of 10 molecules or less per PCR reaction. Assays must pass clinical validation with the assay being tested against clinical samples characterized as positive or negative by another acceptable method. These methods will vary depending on diagnostic methodologies available for the organism of interest and include IDEXX BioResearch conventional PCR assays, sequence analysis, microbiologic methods (i.e., Vitek, MALDI-TOF), microscopic examination or ATCC deposited isolates. Sequence analysis is performed on select positive samples to confirm amplification of the intended target. The assay passes the validation phase if it is proven to be sensitive and specific for the intended target(s).
